# Vancomycin dosing in neonates: enhancing outcomes using population pharmacokinetics and simulation

**DOI:** 10.3389/frabi.2025.1568931

**Published:** 2025-05-08

**Authors:** Sílvia M. Illamola, Jiraganya (JJ) Bhongsatiern, Angela K. Birnbaum, Shaun S. Kumar, Joshua D. Courter, David B. Haslam, Karel Allegaert, David M. Reith, Pankaj B. Desai, Catherine M. Sherwin

**Affiliations:** ^1^ Department of Experimental and Clinical Pharmacology, College of Pharmacy, University of Minnesota, Minneapolis, MN, United States; ^2^ Department of Pharmaceutical Sciences, University of Cincinnati, Cincinnati, OH, United States; ^3^ Quantitative Pharmacology, Regeneron Pharmaceuticals, Tarrytown, NY, United States; ^4^ Division of Clinical Pharmacology, Department of Pediatrics, University of Utah School of Medicine, Salt Lake City, UT, United States; ^5^ Clinical Pharmacology Modeling Simulation, Parexel International, Sydney, NSW, Australia; ^6^ Pharmacy, Cincinnati Children’s Hospital Medical Center, Cincinnati, OH, United States; ^7^ Division of Infectious Diseases, Department of Pediatrics, Cincinnati Children’s Hospital Medical Center, Cincinnati, OH, United States; ^8^ Department of Pharmaceutical and Pharmacological Sciences, KU Leuven, Leuven, Belgium; ^9^ Department of Development and Regeneration, KU Leuven, Leuven, Belgium; ^10^ Department of Hospital Pharmacy, Erasmus MC, Rotterdam, Netherlands; ^11^ Office of the Dean, Dunedin School of Medicine, University of Otago, Dunedin, New Zealand; ^12^ Department of Pharmacology and Toxicology, Wright State University Boonshoft School of Medicine, Dayton, OH, United States; ^13^ Internal Medicine, UWA Medical School, The University of Western Australia, Perth, WA, Australia; ^14^ Clinical Pharmacology and Pharmacometrics, Differentia Biotech Ltd., San Francisco, CA, United States

**Keywords:** neonatology, population pharmacokinetics, dosing regimens, simulations, the probability of target attainment, vancomycin

## Abstract

**Introduction:**

Optimizing vancomycin dosing in neonates is a critical yet complex goal. Traditional trough concentration-based dosing strategies correlate poorly with therapeutic efficacy and often fail to account for the significant renal function variability and drug clearance in neonates. The 24-hour area under the concentration-time curve to minimum inhibitory concentration (AUC_24_/MIC) ≥ 400 mg h/L has emerged as a superior pharmacodynamic target. Population pharmacokinetics (PopPK) models allow optimized dosing by incorporating neonatal-specific factors such as postmenstrual age (PMA), gestational age (GA), serum creatinine (SCr), and weight.

**Objective:**

To develop optimized vancomycin dosing regimens for neonates that achieve an 80% probability of target attainment (PTA) for an AUC_24_/MIC ≥ 400 mg h/L across diverse clinical cohorts and simulated neonatal populations.

**Methods:**

Real-world data from three international centers (Belgium, New Zealand, USA), including 610 individuals and 2399 vancomycin concentrations, were used to externally evaluate a previously published PopPK model (NONMEM^®^). Missing data, including body weight, were imputed using Amelia II version 1.7.3 for R, while Zelig for R integrated multiple imputed datasets. A virtual population of 10,000 neonates was independently generated using MATLAB to simulate clinical scenarios considering covariates such as PMA, GA, SCr, body weight, and imputed body length.

**Results:**

Simulations showed that PMA and SCr were key covariates that significantly improved PTA, particularly in preterm neonates. Preterm neonates achieved PTAs of 80% with daily doses of 30 or 40 mg/kg/day, while term neonates required 15 mg/kg every 8 hours or 20 mg/kg every 12 hours. The simulations demonstrated that these optimized dosing strategies achieved an 80% PTA for AUC_24_/MIC ≥ 400 mg h/L in the virtual neonatal population. For neonates with PMA < 29 weeks and SCr > 0.6 mg/dL, including SCr as a covariate increased the likelihood of achieving the target from 65% to 87%.

**Conclusion:**

Incorporating developmental factors like PMA and SCr into vancomycin dosing strategies achieved robust and clinically relevant outcomes. The optimized regimens achieved an 80% PTA for the AUC_24_/MIC target for preterm and term neonates. These findings offer a scalable framework for improving neonatal vancomycin pharmacotherapy across diverse populations and clinical settings.

## 1 Introduction

Vancomycin is an antibiotic of choice for treating methicillin-resistant *Staphylococcus aureus* (MRSA) in neonates (American Academy of Pediatrics, 2009; American Academy of Pediatrics, 2012; American Academy of Pediatrics, 2021). The initial 2009 guideline (Rybak et al., 2009; Liu et al., 2011) by the Infectious Diseases Society of America (IDSA) and American Society of Health-System Pharmacists, primarily focused on adult patients, recommended a 24-hour area under the concentration-time curve to minimum inhibitory concentration (AUC_24_/MIC) ratio ≥ 400 mg h/L and trough concentrations of 15–20 mg/L for the treatment of severe invasive MRSA infections (Rybak et al., 2009). While these guidelines were widely adopted, their applicability to neonatal and pediatric populations remained uncertain, necessitating further pharmacokinetic investigations. An increased incidence of nephrotoxicity associated with vancomycin trough concentrations > 15 mg/L led to the 2020 updated guidelines (Rybak et al., 2020), which no longer recommend the use of trough concentrations as a target. Instead, recommendations changed to AUC24/MIC of 400–600 mg h/L for adults, and AUC_24_/MIC of 400 mg h/L (potentially up to 600 mg h/L) for children ages 3 months and older, for MRSA with a MIC of ≤ 1 mg/L. The new target has been proven to achieve maximum efficacy while minimizing nephrotoxicity (Rybak et al., 2020).

In neonates, the efficacy target of AUC_24_/MIC is established at 400 mg h/L, but there is no clear evidence of the relationship between the upper limit and nephrotoxicity (Tang et al., 2021). In neonates, antibacterial therapy is complicated by developmental changes inherent to this population, reflected in extensive interindividual variability of its pharmacokinetic parameters (Jarugula et al., 2022). As no specific vancomycin toxicity exposure target has been established in neonates, it is challenging to rationally optimize antibiotic dosing strategies in neonates to improve pharmacodynamic target attainment (Pham, 2020). However, to date, several studies have used population pharmacokinetic models (PopPK) to evaluate the probability of achieving the recommended AUC_24_/MIC target using recommended vancomycin dosing regimens in pediatric populations (Madigan et al., 2015). These models have provided valuable insights into optimizing vancomycin therapy, particularly in challenging cases (Hughes et al., 2023). As a result, high daily doses of ≥60 mg/kg have been suggested to achieve the AUC_24_/MIC ≥400 target in specific pediatric populations where standard regimens might fall short. However, the implications of such higher doses raise important considerations, particularly in balancing efficacy with the risk of nephrotoxicity, underscoring the need for individualized dosing strategies based on robust pharmacokinetic models (Frymoyer et al., 2009; Frymoyer et al., 2010; Le et al., 2013). Compared to prior PopPK studies (Madigan et al., 2015; Pham, 2020; Jarugula et al., 2022), our study provides a comprehensive external validation of an existing vancomycin model across three independent neonatal datasets, ensuring robust generalizability. Additionally, unlike previous models that primarily focus on retrospective data analysis, we implement a prospective simulation approach to optimize neonatal vancomycin dosing. Our study also uniquely evaluates the impact of different renal function estimates on model performance, further refining neonatal dosing strategies.

The current study focuses on advancing neonatal pharmacotherapy by refining dosing strategies, and transitioning from traditional approaches and shifting from peak and trough (Cmin)-based targets to an approach optimized for AUC_24_/MIC. These approaches are better indicators of therapeutic efficacy. While traditional models often emphasize achieving specific concentration thresholds, tools (Frymoyer et al., 2019) such as population-based dosing models can be used for optimized dosing strategies tailored to the unique physiological and pharmacokinetic characteristics of neonates. This study aimed to establish a population-based dosing regimen that ensures optimal exposure-response relationships. Previous studies have identified two main difficulties when applying PopPK models in clinical practice, particularly determining the optimal dosing regimen for neonates: the generalizability of models to different patient groups and how organ maturation alters pharmacokinetics (Janssen et al., 2016; Germovsek et al., 2019). In these studies, the vancomycin dose and interval have been adjusted based on growth and development changes to ensure that high rates of probability of target attainment (PTA) can be reached in newborn infants (Rodvold et al., 1997). These prior studies on neonatal dosing have also often identified flaws in Cmin-based approaches (Nelson and Bradley, 2012), including a poor match with pharmacodynamic targets and failure to capture the dynamic physiological shifts that characterize neonates. This is particularly critical for neonates, whose renal clearance vary significantly due to rapid maturation processes. By incorporating important individualized variables like postmenstrual age (PMA), gestational age (GA), serum creatinine (SCr), and current weight into the dosing algorithm, the approach proposed here allows for a more accurate estimate of drug clearance and exposure. This relatively novel methodology represents a significant refinement in the application of PopPK and model-based simulations to optimize vancomycin dosing in neonates. While similar approaches have been explored in pediatric pharmacotherapy, this study integrates a large-scale virtual neonatal population, advanced covariate stratification, and external validation across multiple clinical datasets, providing a more robust and clinically applicable dosing framework.

In neonates, several dosage schedules consider clinical factors, population demographics, neonatal age (American Academy of Pediatrics, 2009) and weight (Hey, 2011; Young and Mangum, 2011; Ainsworth, 2014). Interestingly, some dosing regimens are not necessarily designed to represent the whole age spectrum of the neonatal population because they were created in cohorts of uniformly generated (sub)populations or limited sample sizes of patients with specific characteristics. Large representative newborn cohorts are readily available, allowing for robust modeling of virtual preterm and term infants with the potential full range and realistic distributions of their clinical traits (Pomerantseva and Ilicheva, 2012; Hughes et al., 2023). This study aimed to use generated virtual populations to provide an unbiased evaluation of vancomycin dosing regimens by using a previously published model (Bhongsatiern et al., 2015). The goal was to assess whether at least 80% of subjects achieved an AUC_24_/MIC of 400 mg h/L, thereby optimizing dosing strategies at the population level.

## 2 Methods

### 2.1 Population pharmacokinetic model

All pharmacokinetic analyses in this study were conducted using a previously published PopPK model (Bhongsatiern et al., 2015), based on data from neonatal patients (n=152) with late-onset sepsis, which includes HT as a covariate for estimating glomerular filtration rate (eGFR). While other vancomycin PopPK models for neonates exist, some of which do not require HT as a covariate, the selected model was chosen due to its robust external validation, inclusion of key maturational covariates (PMA, weight, and SCr), and its demonstrated predictive accuracy in neonatal populations. This model has been evaluated across multiple external datasets and provides a well-characterized framework for dose optimization.

The dataset used in the published PopPK model was derived from clinical records and included both preterm and term neonates who had a minimum of one vancomycin concentration measurement and one positive microbiological culture. Given that the data originated from real-world clinical settings, it underwent thorough cleaning and validation processes to ensure accuracy and consistency. This step involved querying and reconciling inconsistencies, such as missing or implausible values, to establish a robust dataset for modeling (Pomerantseva and Ilicheva, 2012).

The published PopPK model referenced in this study includes structural pharmacokinetic parameters described by established equations (Bhongsatiern et al., 2015). However, the focus of this study is not on redeveloping or validating the existing PopPK model but rather on leveraging it to evaluate and optimize vancomycin dosing regimens in neonates.

In the following, the model will be referred to as “the published model.”

This study integrates two complementary methodologies: (1) External validation of a previously published PopPK model using NONMEM, and (2) Optimization of vancomycin dosing regimens through Monte Carlo simulations performed in MATLAB. The use of NONMEM ensures that the PopPK model accurately predicts vancomycin pharmacokinetics across three independent clinical datasets. However, to explore optimal dosing strategies and improve PTA beyond existing models, we employed MATLAB for large-scale Monte Carlo simulations (section 2.2.2), allowing for flexible AUC24 calculations across a virtual population of 10,000 neonates. MATLAB was chosen over NONMEM for Monte Carlo simulations due to its superior computational efficiency for large-scale simulations (10,000 neonates) and flexible implementation of PTA calculations. Unlike NONMEM, which optimizes individual PK parameters for each subject iteratively, MATLAB allows for parallelized computations, enabling faster simulation processing. This decision aligns with prior pharmacometric studies utilizing MATLAB for similar large-scale dosing evaluations (Wang et al., 2015; Mavroudis et al., 2019; Stader et al., 2019). This approach ensures that simulations reflect a clinically representative range of neonatal characteristics while enabling efficient evaluation of multiple dosing regimens.

### 2.2 Study population

For this investigation, three clinical datasets that were acquired from various academic institutions were used. The study was open to neonatal patients with PMA up to 44 weeks and at least 1 concentration(s) of vancomycin. **Table 1** summarizes the three datasets. Dataset 1 was separately collected and excluded patients already included in the published model (Bhongsatiern et al., 2015). The institutions in the listed countries applied for and received a waiver of consent from the various ethical committees in order to analyze these retrospective de-identified datasets.

**Table 1:** Characteristics for all datasets.

Characteristics	Dataset 1	Dataset 2	Dataset 3
Setting	Salt Lake City, USA^1^	Leuven, Belgium^2^	Dunedin, New Zealand^3^
Time frame	01/01/2006 - 08/23/2012	06/01/2011 - 06/01/2012	10/23/2000 - 11/29/2007
Number of individuals (N)	403	185	22
Vancomycin interval administration (hours) (range)	8 - 18	8 - 12	24
Vancomycin dose (mg/Kg/day) (mean ± SD)	30.06 ± 14.00	28.84 ± 10.28	18.71 ± 4.75

^1^From Intermountain Healthcare’s computerized medical records, Department of Pediatrics, Division of Clinical Pharmacology, University of Utah, USA; ^2^Neonatal Intensive Care Unit at University Hospitals Leuven, Belgium; ^3^Department of Women’s and Children’s Health at University of Otago, Dunedin, New Zealand.

Vancomycin was intravenously administered over 60 minutes at dosing intervals between 8 to 24h and doses from 10 to 40 mg/Kg/day (**Table 1**), and blood samples were collected within 0.5 hours of the vancomycin dose (trough) and within 2 hours of the infusion reaching steady state (peak). However, in the Dunedin dataset blood samples were collected within the first 1–2 dosages (first 48 hours). Vancomycin concentrations were measured using various immunoassay-based methodologies (Vandendriessche et al., 2014; Bhongsatiern et al., 2015), all of them with a lower limit of quantification of 2.0 mg/L. These methods were used across all three clinical datasets to ensure consistency in concentration measurements. The Jaffe method (Peake and Whiting, 2006; Allegaert et al., 2012) [Salt Lake City (Bhongsatiern et al., 2015) and Leuven datasets] (Vandendriessche et al., 2014), and an enzymatic assay (Dunedin dataset) were both used to assess the SCr, with lower limit of quantification of 0.2 mg/dl and 0.1 mg/dL for the Jaffe and the enzymatic assay, respectively (Hessey et al., 2017; Roy et al., 2019). The values of SCr from both methods were converted to equivalent values using a linear regression equation proposed by Srivastava et al (Srivastava et al., 2009). With a maximum time difference of 24 hours, the SCr value that was closest to a vancomycin concentration was selected for the study. If all SCr values available exceeded 24 hours, the SCr value was imputed to a value of 0.5 mg/dL. This method is commonly used in pediatric studies for missing SCr values (Siew et al., 2013).

#### 2.2.1 Imputation of body length

Body length [height (HT)] is a key factor in calculating creatinine clearance (CrCL) using the modified (“bedside”) Schwartz equation (Schwartz et al., 2009), which in turn is critical for estimating vancomycin clearance in neonates (Hessey et al., 2017). However, in this study, HT data were missing for a significant proportion of patients, particularly in some datasets where HT data were not recorded at all. Since accurate dosing depends on precise estimation of CrCL, it was necessary to impute missing length data to ensure the validity of the pharmacokinetic model.

The HT data in the Salt Lake City dataset were insufficient, with approximately 6.5% of HT data missing. In the Leuven and Dunedin datasets, HT records were entirely absent (100% missingness). HT was required for CrCL computation, therefore multiple imputations were performed using Amelia II version 1.7.3 for R and the expectation-maximization with bootstrap approach (Pilowsky et al., 2024) to ensure accurate estimation of eGFR for vancomycin clearance calculations. This approach was chosen due to its ability to handle continuous and categorical variables and preserve underlying relationships among variables (Honaker et al., 2011). Observed HT data from the Salt Lake City dataset were imputed to account for partial missingness, while imputations for the Leuven and Dunedin datasets relied on other available factors (e.g., weight, SCr, PMA, GA, and postnatal age (PNA)) to predict HT values (Honaker et al., 2011). HT is a critical parameter that correlates with other demographic and physiological variables. To mitigate potential bias, strategies were employed, including variables strongly correlated with HT, conducting the imputation process separately for subgroups based on sex and GA categories, generating multiple imputed datasets, and performing sensitivity analyses to compare results obtained using the imputed dataset with those from a complete-case analysis. The distributions of these factors and their multiple imputations maintained their relationships, ensuring accurate HT prediction despite the complete absence of observed HT in the Leuven and Dunedin datasets. The imputed data were assessed for biological plausibility, and distributions were compared to reference neonatal growth standards to minimize bias. While the method used effectively addressed missing data, we acknowledge that using a PopPK model that do not require HT as a covariate could potentially reduce reliance on imputation in future studies. Zelig for R (Choirat et al., 2017) was used to integrate the imputed datasets and conduct statistical analysis.

#### 2.2.2 Simulation of the virtual population

A virtual population of neonates (n=10,000) was generated to simulate various clinical scenarios. To capture the full spectrum of neonatal characteristics, including GA, PMA, and body weight, we used real-world data distributions and reference values from WHO child growth standards (WHO Multicentre Growth Reference Study Group, 2006) and published literature (Ceriotti et al., 2008; Boer et al., 2010). A modified version of the Expectation-maximization algorithm was used to estimate missing covariates and ensure internal consistency among PMA, GA, weight, and SCr. This approach preserved the relationships among these variables, ensuring that the simulated neonatal population reflected real-world variability in pharmacokinetics. Weight, SCr, PMA, GA, and PNA were used as covariates from the model-building dataset along with all three datasets. In order to examine the maximum likelihood for each bin, distribution analyses of the mean and covariance were completed and added. Thirdly, using equations that expressed the relationship between the newborn age (PMA = (PNA/7) + GA) and significant correlations among weight, SCr, and HT, covariates that described preterm (n=5,000) and term (n=5,000) neonates were separately simulated. MathWorks, Inc., Natick, Massachusetts’ MATLAB and statistics Toolbox Release 2013a was used to run the simulations.

The study uses the World Health Organization (WHO Multicentre Growth Reference Study Group, 2006) growth standards as a reliable reference for assessing growth and developmental parameters across diverse populations. These standards provide normative data on weight, length, and other key growth metrics stratified by age and sex, enabling accurate characterization of neonates at different developmental stages. The study ensures that dosing strategies developed are aligned with universally recognized benchmarks, enhancing their applicability across various clinical settings and populations. The WHO standards account for variations in growth patterns due to different geographical and socioeconomic factors, generating representative and inclusive dosing recommendations. The simulation approach aligns with recent advancements in neonatal pharmacokinetics, as outlined by Alsultan et al (Alsultan et al., 2023), who demonstrated the utility of simulation techniques in optimizing vancomycin dosing in very low birth weight neonates. This large sample size allows for robust simulation of different clinical scenarios and patient subgroups, including preterm and term neonates, ensuring the results are generalizable to the broader neonatal population. The large virtual population size also strengthens the statistical power of the analysis and minimizes potential biases.

### 2.3 Model evaluation

PDx-Pop version 5.2 (ICON Development Solutions, Ellicott City, MD) interfaced with nonlinear mixed-effect modeling version 7.3 (NONMEM^®^) was used to externally evaluate the generalizability of the published model against all three datasets. Using the command NONMEM MAXEVAL = 0, POSTHOC (Sheiner and Beal, 1981), population and individual projected concentrations were obtained from the published model 1.

Individual predictions (IPRED) were generated using a *post hoc* estimation approach, where all available observations were used to fit PK parameters and then predict each observation individually. The model did not use an iterative prediction approach (i.e., using the first K observations to predict the (K+1)th observation); instead, it leveraged all available data points simultaneously to optimize parameter estimation. This approach ensures robust individual predictions while maintaining consistency across the dataset.

We first compared goodness-of-fit plots of the population predicted concentrations and the observed concentrations to assess predictive performance of the model. We estimated the population predictions (PRED) and compared to the observed concentrations using the prediction error (PE%):


$$PE=\frac{Observed-PRED\;or\;IPRED}{Observed}$$

To assess the predictive performance of the externally validated PopPK model, we used the following standard validation criteria: median prediction error (MDPE) and median absolute prediction error (MAPE) as a measure for model bias and precision, respectively; F20 (PE% within ± 20%) and F30 (PE% within ± 30%) for accuracy and precision, respectively. To consider a model acceptable, MDPE should be ≤ ± 20%, MAPE ≤ ± 30%, F20 ≥ 35%, and F30 ≥ 50% (Mao et al., 2018; Cai et al., 2020). These metrics ensure that the model accurately captures the variability in vancomycin pharmacokinetics across different neonatal.

Visual predictive check (VPC) (Karlsson and Savic, 2007) and normalized prediction distribution errors (npde) (Brendel et al., 2006) were simulation-based diagnostic techniques used for model evaluation (Sherwin et al., 2012). The published model (Bhongsatiern et al., 2015) estimated parameters were used to simulate all three datasets (n=1000). Simulated concentrations at the 50th, 5th, and 95th percentiles were displayed against dose-related time in hours for the VPC. It was anticipated that 90% of the observed data points would fall within the predicted range of 5th to 95^th^ intervals. The distribution of the npde, which was computed using NONMEM, was predicted to follow a *N* (0, 1) distribution, as seen by its mean and variance.

### 2.4 Monte Carlo simulation

MATLAB version 2013a was used to evaluate published dosing schedules and optimize a new dosing strategy, using two methods—global inclusion (no population stratification) and covariate-based stratification. Monte Carlo simulations, as detailed in published literature (Mehrotra et al., 2012; Alsultan et al., 2023), provide robust frameworks for dosing optimization by integrating patient-specific variables and pharmacokinetic variability. Briefly, the simulation process followed these steps: (1) Virtual Population Generation: A clinically relevant neonatal population was created using real-world distributions of PMA, WT, and SCr (for CrCL calculations); (2) Time-Concentration Profile Simulations: The previously published PopPK model equations were implemented in MATLAB to simulate drug concentration-time profiles for each neonate; (3) AUC_24_ Calculations: AUC was computed using log-linear trapezoidal integration, ensuring a more precise representation of exposure than simple trapezoidal methods; (4) Covariate-Based Optimization: PTA calculations were stratified by key covariates (WT, PMA, CrCL) to identify optimal dosing regimens. Using the pharmacokinetic parameters calculated from the published model (Bhongsatiern et al., 2015), concentration-time profiles were simulated to determine vancomycin exposure (i.e., AUC). Following the intermittent brief infusion paradigm with a 1-hour infusion time, vancomycin concentrations were simulated as follows:


$$C_{preinf}=\;\left[\frac{dose}{\left(CL\right)\left(tk0\right)}\right]\left(1-exp^{-k_{el}\left(t-dT\right)}\right)$$

$$C_{postinf}=\;\left[\frac{dose}{\left(CL\right)\left(tk0\right)}\right]\left(1-exp^{-k_{el}\left(tk0\right)}\right)\left(exp^{-k_{el}\left(t-dT-tk0\right)}\right)$$

Where, tk0, duration of infusion (hr.), kel, elimination rate constant (hr-1), dT, dosing time, which is an array of times at which doses are given (hr.), and t, time, which is a discrete set of points detailing the entire dosing profile (hr.), indicated by the concentrations before and after the end of the infusion (Cpreinf and Cpostinf, respectively).

A simulated-time matrix, which represents the time indices at which the infusion rate started and terminated (1–96 hr.), was used to calculate the minimum (Cmin or trough) and maximum (Cmax or peak) concentrations (mg/L). For a 24 hour AUC segment (72–96 hr.), the steady-state AUC (mg.hr/L) was calculated using the trapezoidal rule, as provided by $AUC^{t_{2}-t_{1}}=\;\frac{C_{p}^{t1}+\;C_{p}^{t2}}{2}\left(t_{2}-\;t_{1}\right)$ , where Cp stands for plasma concentrations and t2-t1 is the period for the segment from Cp1 to Cp2. AUC_24_ calculations were performed using the log-linear trapezoidal integration method, rather than direct integration from the model. This approach was selected due to its ability to provide robust and flexible exposure estimations across multiple dosing regimens, particularly when dealing with sparse or variable sampling time points. The log-linear trapezoidal method balances accuracy with computational efficiency, making it suitable for large-scale Monte Carlo simulations in this study.

The dosing regimens listed in **Table 2** were used to optimize a new dosing strategy. Therapeutic target was defined as reaching steady-state AUC_24_ ≥ 400 mg.hr/L at the MIC 1 g/mL, which aligns with the lower bound for efficacy. However, this study does not explicitly mention assessing a maximum AUC or a defined upper limit for nephrotoxicity prevention. The PTA of 80% was used as a predictor of effective treatment. Vancomycin dosages ranging from 10 to 40 mg/kg/day at dosing intervals of 8, 12, and 24 hours were used to simulate concentration-time profiles for dosing optimization.

**Table 2:** Vancomycin dose and frequency of administration of the different sites.

Dosing Regimens	Characteristics of the hypothetical population			Dose (mg/kg)	Interval (hrs.)
PMA and PNA-based dosing (Young and Mangum, 2011)	PMA (weeks)	GA (weeks)	PNA (days)		
	≤29 30-36 37-44		0-14 >14 0-14 >14 0-7 >7	10 10 10 10 10 10	18 12 12 8 12 6
PMA-based dosing (Hey, 2011)	<29 29-35 36-44			15 15 15	24 12 8
PMA-based dosing (Ainsworth, 2014)	≤29 30-33 34-37 38-44			20 20 20 15	24 18 12 8
Renal function-based dosing (Capparelli et al., 2001; American Academy of Pediatrics, 2012; Nelson and Bradley, 2012)	SCr (mg/dL)	GA (weeks)	PNA (days)		
	>1.4 1.1-1.4 0.8-1 0.5-0.7 <0.5	≤28	≤60	15 10 15 20 15	48 24 24 24 12
	>1.6 1.3-1.6 1-1.2 0.7-0.9 <0.7	>28	≤60	15 10 15 20 15	48 24 24 24 12
			>60	10 10	8 6
Weight-based dosing (American Academy of Pediatrics, 2009)	CWT (kg)	GA (weeks)	PNA (days)		
	<1.2 1.2-2 >2		<7	15 10 15 10 15	24 12 18 8 12
	<1.2 1.2-2 >2		≥7	15 10 15 10 15	24 8 12 6 8

CWT, current body weight (kg); SCr, serum creatinine concentration (mg/dL); PMA, postmenstrual age (weeks); GA, gestational age (weeks); PNA, postnatal age (days).

## 3 Results

### 3.1 Study populations

#### 3.1.1 Data comparison

Demographic and clinical traits of all three datasets were compared (**Table 3**). A total of 2399 vancomycin concentrations were incorporated into the analysis, distributed as follows: 345 concentrations from Salt Lake City dataset, 1685 concentrations from Leuven dataset, and 369 concentrations from Dunedin dataset. A 0.33% (n=7 from Leuven dataset, and n=1 from Dunedin dataset) of vancomycin concentrations were below the limit of quantification (BLOQ). As BLOQ were < 1%, for modeling and analysis, BLOQ values were replaced with 1.0 mg/L (LLOQ/2) (Beal, 2001; Irby et al., 2021). In all three datasets, preterm neonates made up more than 70% of the patients, with the Dunedin dataset having the highest percentage of preterm patients (100%). The median (interquartile range (IQR)) GA was 30 weeks (26–37) for Salt Lake City dataset, and 32 weeks (28–37) and 25 weeks (24–28) for Leuven and Dunedin datasets, respectively; the median (IQR) of PNA was 13 days (8–21) (Salt Lake City dataset), 8 days (5–13) (Leuven dataset), and 14 days (10–17) (Dunedin dataset). The median (IQR) of PMA was 33.4 weeks (28.4–38.9) for Salt Lake City dataset, and 34 weeks (30–39) and 27.4 weeks (26.2–30.0) for Leuven and Dunedin datasets, respectively. The Leuven and Dunedin datasets did not contain any records for HT. The Salt Lake City dataset did not require any imputation for SCr values. In contrast, the Leuven and Dunedin datasets required imputation for 6 (3.24%) and 7 (31.8%) of SCr values, respectively, setting them to 0.5 mg/dL. The median (IQR) of SCr for the Salt Lake City and Leuven datasets was similar 0.50 (0.40-0.69) mg/dL and 0.49 (0.39–0.62) mg/dL, respectively, whereas the Dunedin dataset had a higher median SCr of 0.63 (0.48–0.96) mg/dL. The higher SCr values in the Dunedin dataset corresponded with the lower mean PMA in that population, which was used to model the virtual population. In **Table 3**, specific patient demographics are enumerated.

**Table 3:** Demographics and laboratory characteristics for all datasets.^†^

Characteristics	Dataset 1 (n=403)	Dataset 2 (n=185)	Dataset 3 (n=22)
Preterm (Gestational age <37 weeks, %)	73.7	70.8	100
Term (%)	26.3	29.2	0
Apgar 1 minute	4 (0-7)	N/A	N/A
Apgar 5 minute	7 (0-9)	N/A	N/A
Multiple gestations (%)	13.2	N/A	N/A
Male (%)	60.3	51.4	54.5
Female (%)	39.7	48.6	45.5
Birth weight (kg)	1.38 (0.81-2.82)	1.78 (1.13-2.68)	0.74 (0.69-0.85)
Body weight (kg)	1.64 (1.0-2.84)	1.84 (1.15-2.85)	0.95 (0.90-1.24)
Body Length (cm)	41 (35-48)	N/A	N/A
Gestational age (weeks)	30 (26-37)	32 (28-37)	25 (24-28)
Postnatal age (days)	13 (8-21)	8 (5-13)	14 (10-17)
Postmenstrual age (weeks)	33.4 (28.4-38.9)	34 (30-39)	27.4 (26.2-30.0)
Serum vancomycin concentration (mg/L)	21.1 (4.1 - 46.6)	8.9 (1.0 - 37.8)	11.7 (1.3 - 86.0)
Serum creatinine concentration (mg/dL) ^§^	0.50 (0.40-0.69)	0.49 (0.39-0.62)	0.63 (0.48-0.96)
Creatinine clearance (mL/min/1.73 m^2^) ^‡^	33.9 (22.2-47.7)	33.1 (23.9-50.3)	24.9 (15.0-28.3)

Dataset 1: Salt Lake City; Dataset 2: Leuven; Dataset 3: Dunedin.

^†^Baseline characteristics are reported as median (interquartile range) unless specified otherwise. Dataset2 was reported in (Vandendriessche et al., 2014).

^§^Serum creatinine concentrations in Dataset 3 were measured using the Jaffe method and were converted to an enzymatic method equivalent using a linear equation described by Srivastava et al (Srivastava et al., 2009). Converted values are presented in the current table.

^‡^Calculated using the modified Schwartz equation (CrCL=0.413*HT/SCr) (Schwartz et al., 2009).

N/A, not available; Apgar, a score to evaluate a newborn’s physical condition based on Appearance, Pulse, Grimace, Activity, and Respiration; HT, body length (cm); SCr, serum creatinine concentration (mg/dL).

#### 3.1.2 Imputation of body length

The imputation of HT successfully substituted missing data for all three datasets. There was a positive correlation between the entire PMA range and the observed and imputed HT in the Salt Lake City dataset, as well as with the imputed HT in the Leuven dataset (**Supplementary Figure S1**). However, in the Dunedin dataset, the imputed HT were only correlated with PMA ranges of 26.2 to 30.0 weeks (**Supplementary Figure S1**). In the Leuven dataset, the distributions of the imputed HT (mean ± SD = 41.5 ± 8.0 cm, median [IQR] = 40.9 [35.9-47.50] cm) were similar to those of the Salt Lake City observed; however in Dunedin dataset, the distributions of the data (mean ± SD = 34.4 ± 3.8 cm, median [IQR] = 33.5 [31.8-36.5] cm) were pushed to the lower range.

#### 3.1.3 Simulation of the virtual population

The study uses simulated dosing scenarios to provide optimized recommendations for vancomycin pharmacokinetics and target attainment in neonatal patients. The large population size ensures accurate predictions across different subpopulations, making the findings relevant to a wide range of patients. The virtual population model accurately represents real-world neonatal populations, considering growth patterns, renal function, and developmental trajectories (Ceriotti et al., 2008). However, the datasets used in this study primarily represent neonatal populations from high-income countries, which may not fully capture the diversity of neonatal characteristics seen in resource-limited settings. Factors such as low birth weight, under nutrition, and varying rates of prematurity can significantly impact vancomycin pharmacokinetics.

**Figure 1:**
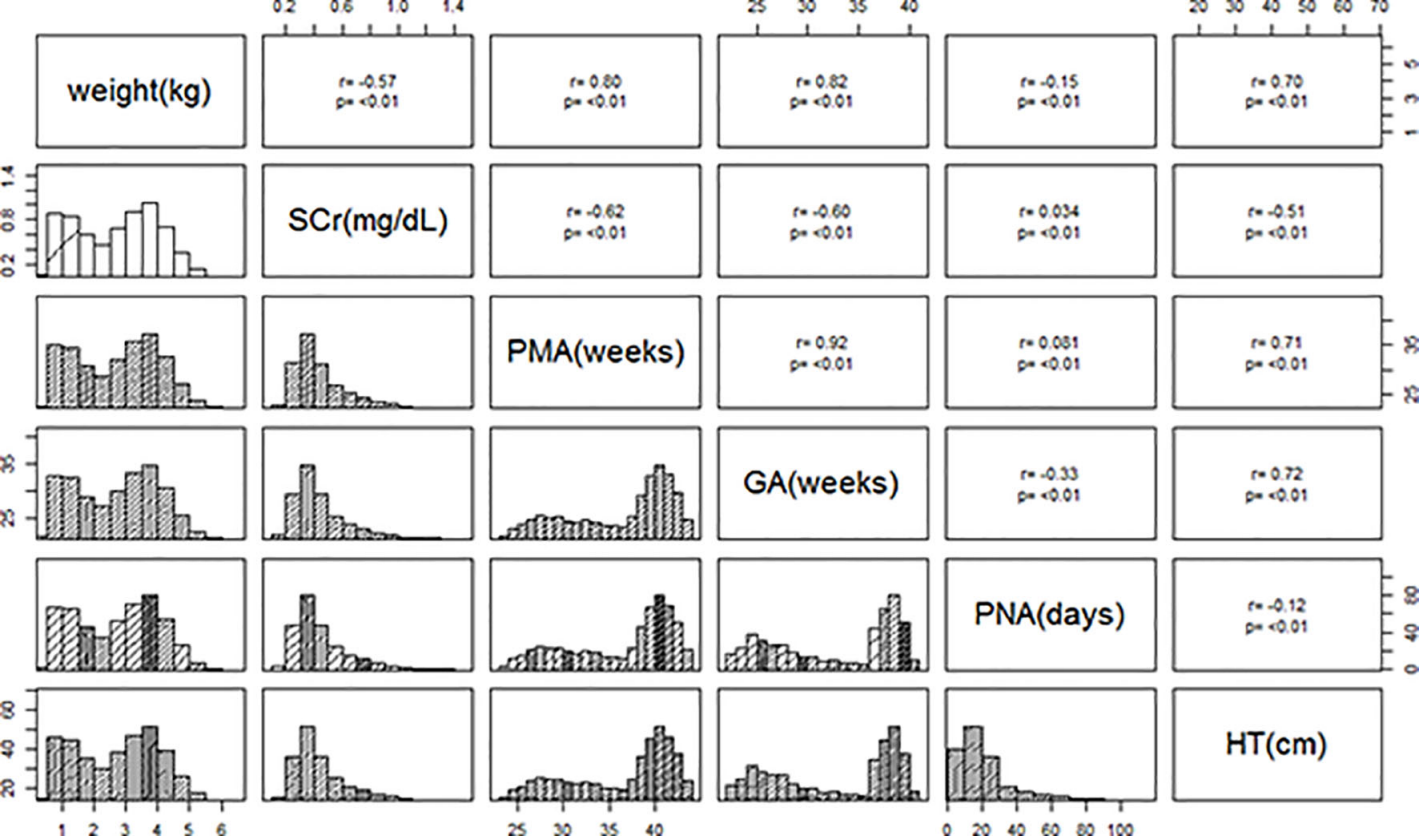
Histograms of covariates of hypothetical preterm and term neonates. Histograms demonstrated the distributions of weight, SCr, HT, PMA, GA, and PNA in the hypothetical preterm (n=5,000) and term (n=5,000) neonates. The correlation coefficients with p-values were also included, indicating well-conserved correlations among covariates. The complete statistical summary was described in **Supplementary Table S1**.

Weight, SCr, PMA, GA, PNA, and HT distributions in preterm (n=5,000) and term (n=5,000) neonates were similar to those in clinical cohorts and reference values. While most variables in the term data were normally distributed, the distributions of covariates in the preterm population were right-skewed, with the exception of PMA (**Figure 1**). **Supplementary Table S1** provides a summary of the full statistical description of the virtual population.

### 3.2 External evaluation

**Figure 2:**
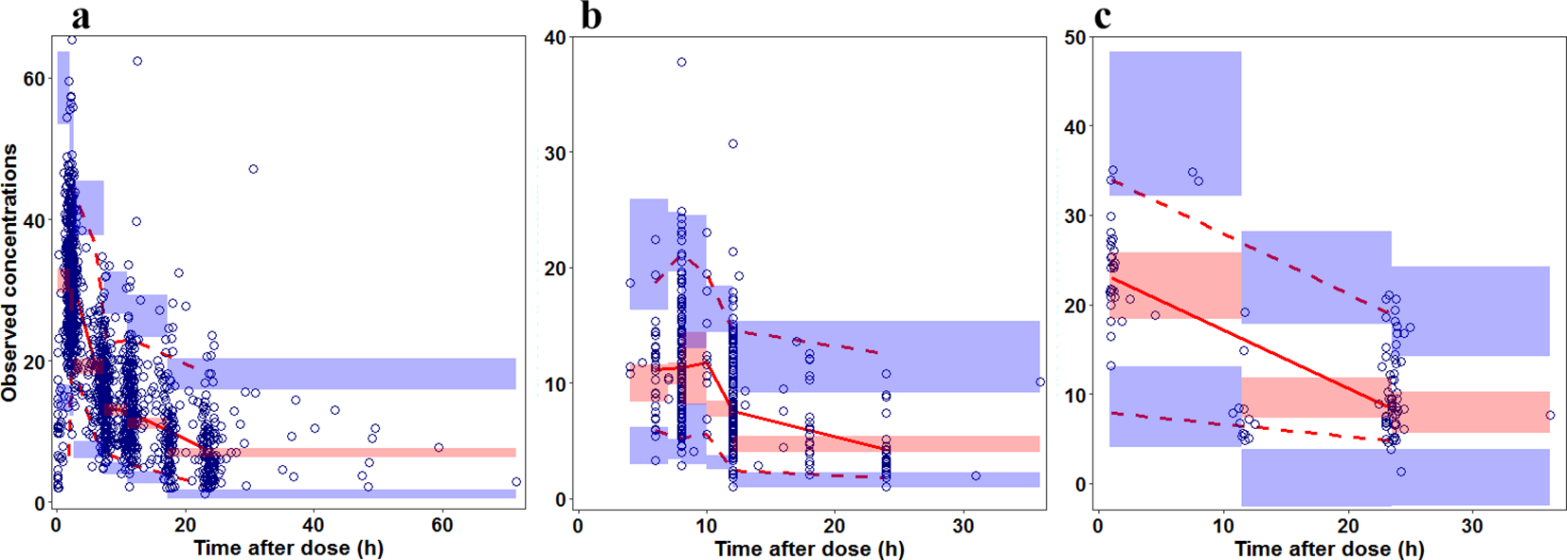
Visual predictive check (VPC) plots of external evaluation datasets 1–3. External evaluation of the population pharmacokinetic model (n=1000) using external datasets (**a**: Salt Lake City dataset, **b**: Leuven dataset, **c**: Dunedin dataset). Observed concentrations were plotted using an opened circle. The dashed lines represent the 5th and 95th percentiles, and the solid line represents the 50th percentile of the observations. Shaded areas are the corresponding model-based 95% confidence intervals for the median and the prediction intervals.

Graphically, the model showed adequacy between predicted and observed concentrations (**Supplementary Figure S2**). The VPC plots of all three datasets show that 6.2%, 10.6%, and 7.5% of the observed data were beyond the 90% prediction interval, respectively (**Figures 2a–c**). For all three datasets, the mean (variance) for the npde was 0.07 (0.845) -0.04 (0.974), and 0.05 (0.346) based on a *N* (0,1) distribution. MAPE values were similar across datasets (~15%), indicating that predictions deviated by an average of 15% from observed concentrations. MDPE values suggested slight underprediction in the Otago (-1.55%) and Utah (-2.23%) datasets, while the Leuven dataset exhibited minimal bias (0.33%). The proportion of predictions within 20% of observed values (F20) ranged from 70.83% to 72.42%, whereas approximately 88% of predictions fell within 30% of observed values (F30) across all datasets (**Table 4**). These findings suggest that the model demonstrates reasonable predictive accuracy, though minor underprediction in some datasets may warrant further refinement if used in the future.

**Table 4:** Predictive performance of the population pharmacokinetic models in the external validation datasets.

	MAPE (%)	MDPE (%)	F20 (%)	F30 (%)
Dataset 1	14.89	-2.23	70.83	88.54
Dataset 2	15.18	0.33	72.42	88.73
Dataset 3	14.71	-1.55	71.21	87.87

Dataset 1, Salt Lake City; Dataset 2, Leuven; Dataset 3, Dunedin.

MAPE (%), mean prediction error; MDPE (%), median absolute prediction error; F20 (%) and F30 (%), percentage of absolute prediction error within 20% and 30%, respectively.

### 3.3 Evaluation of the published dosing regimens

**Figure 3:**
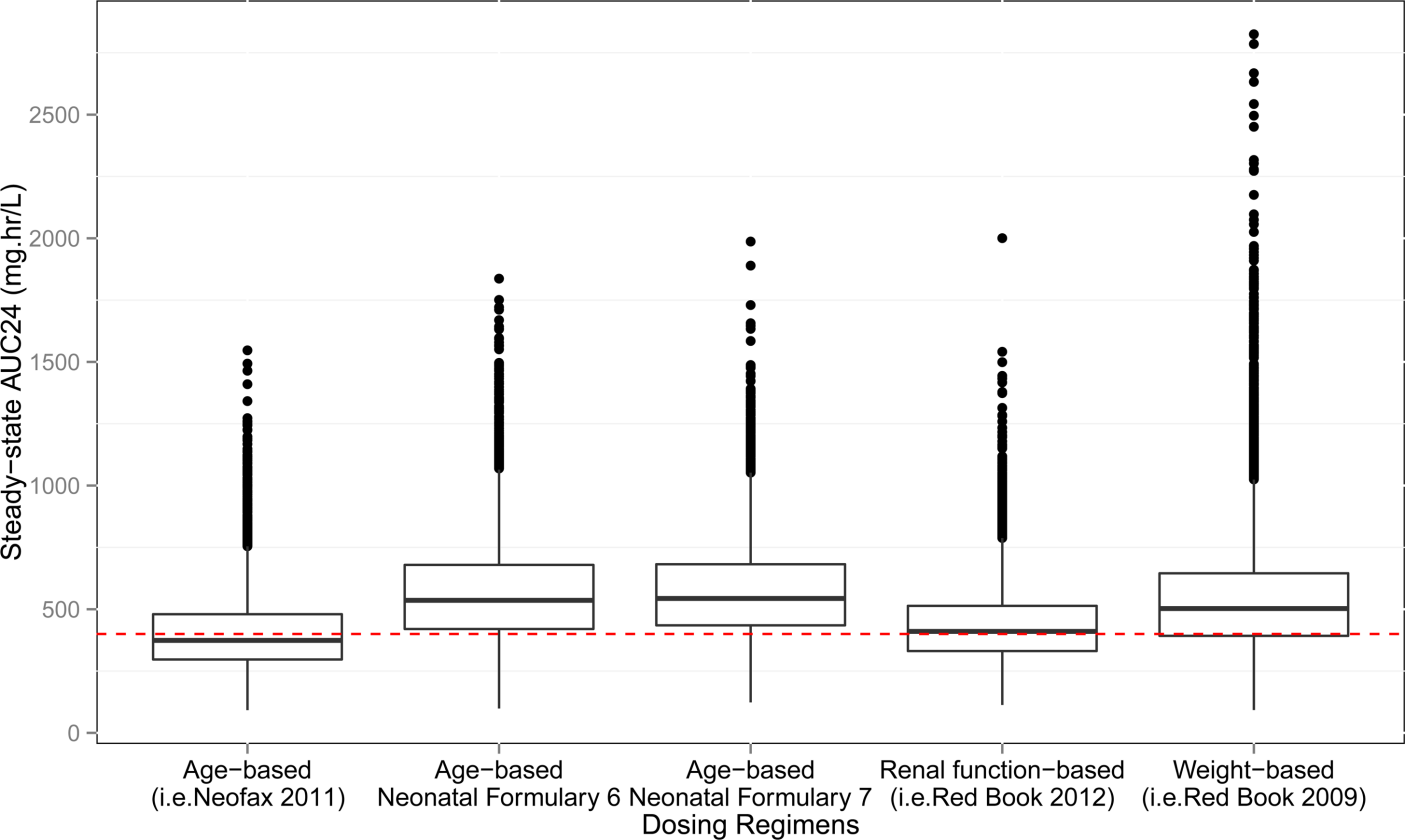
Box plots of steady-state AUC_24_ (mg.hr/L) of the published dosing regimens. Box plots depicting average steady-state AUC_24_ simulated from the published dosing regimens. The red dashed line at 400 mg.hr/L represents the target AUC_24_/MIC ≥400, given MIC value of 1 µg/mL. From left to right: Age-based dosing regimens were PMA and PNA-based from Neofax 2011 (Hey, 2011); Age-based Neonatal Formulary 6 and 7 were PMA-based dosing regimens (Pomerantseva and Ilicheva, 2012; Ainsworth, 2014); Renal function-based dosing regimens were adapted from Red Book 2012 (American Academy of Pediatrics, 2012), Nelson’s Pediatric Antimicrobial Therapy (Germovsek et al., 2019), and Capparelli et al (Johnson et al., 1997); Weight-based dosing regimens were adapted from Red Book 2009 (American Academy of Pediatrics, 2009).

Three dose schedules based on age, one based on renal function, and one based on weight (**Table 1**) were assessed. The evaluation assessed the proportion of the population attaining AUC_24_ ≥ 400 mg·hr/L. In the two PMA only based dosing regimens [the Neonatal Formulary 6th (Hey, 2011) and 7th (Ainsworth, 2014) and the renal function-based dosing regimens (Ringenberg et al., 2015)], about 75% of the virtual population reached the target AUC_24_ ≥ 400 mg.hr/mL, whereas 50% of the population reached the same target in the PMA and PNA-based and the weight-based dosing regimens (**Figure 3**). The established efficacious PTA cutoff of 80% was accomplished by three of the four dose regimens based on PMA-based dosing regimens [Neonatal Formulary 7th (Ainsworth, 2014)] (**Table 5**). However, while the majority of dosing regimens achieve the target exposure range (AUC_24_ ≥ 400 mg.hr/mL), certain higher-dose regimens result in values exceeding the 600 mg h/L toxicity threshold. These findings highlight the potential need for dose refinement, particularly in neonates with impaired renal clearance or other risk factors for drug accumulation.

**Table 5:** Comparison of the probability of target attainment between a published age-based and a proposed dosing strategy.

Sources	Population characteristics		Dosing Regimens		Probability of target attainment
	PMA (weeks)	SCr (mg/dL)	Dose (mg/kg)	Interval (hrs.)	AUC_24_/MIC ≥400
Neonatal Formulary 7	≤29	–	20	24	0.65
Proposed	≤29	0.4-0.6	10 15 20	12 12 24	0.52 0.92 0.52
		>0.6	10 20	12 24	0.89 0.87
Neonatal Formulary 7	30-33	–	20	18	0.84
Proposed	30-33	0.4-0.6	10 15 10	12 12 8	0.33 0.90 0.90
		>0.6	10 15	12 12	0.73 0.99
Neonatal Formulary 7	34-37	–	20	12	0.91
Proposed	34-36	<0.4	20 15 20	12 8 8	0.60 0.71 0.95
		0.4-0.6	10 15 20	8 12 12	0.85 0.81 0.98
		>0.6	10 10 15	12 8 12	0.68 0.94 0.98
Neonatal Formulary 7	38-44	–	15	8	0.86
Proposed	37-44	<0.4	15 20	8 8	0.76 0.96
		0.4-0.6	10 15 20	8 12 12	0.71 0.72 0.96
		>0.6	15 15	12 8	0.99 1.00

Probability of target attainment is from 0 to 1, where 1.00 is the highest probability. Neonatal Formulary 7 was obtained from (Ainsworth, 2014).

PMA, postmenstrual age (weeks); SCr, serum creatinine concentration (mg/dL); AUC24 ≥400, probability of achieving the AUC24 of ≥400 mg.hr/L at MIC of 1 µg/mL.

### 3.4 Optimization of vancomycin neonatal dosing strategies

#### 3.4.1 Determination of vancomycin dose and dosing interval (global inclusion)

**Figure 4:**
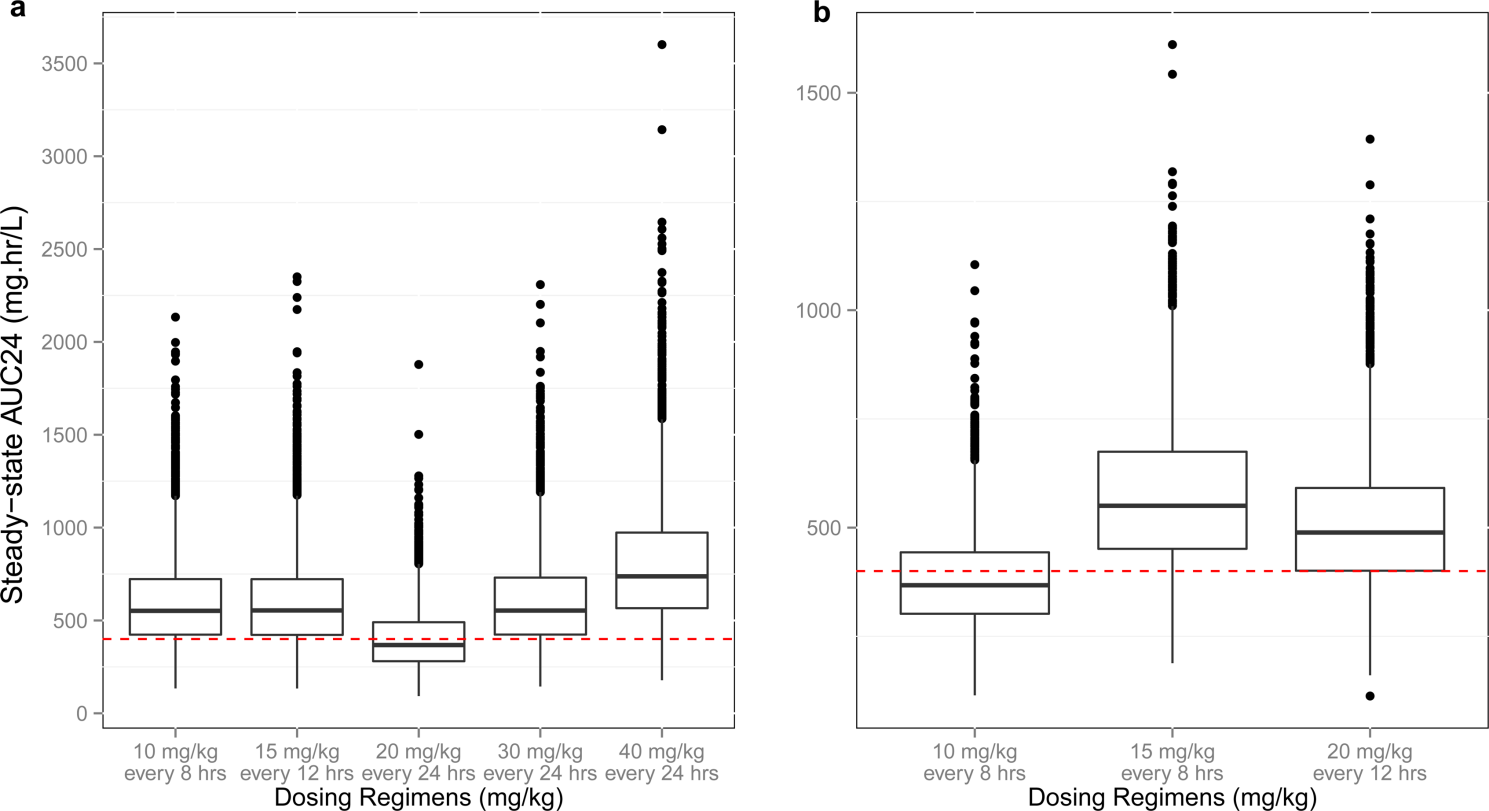
Simulations of steady-state AUC_24_ using vancomycin daily doses of 20–45 mg/kg. Simulations of the average AUC_24_ (mg.hr/L) at the steady state of preterm **(a)** and term **(b)** neonates. Dosing regimens for vancomycin intermittent IV infusion were 10, 15, 20, 30, and 40 mg/kg, and the dosing intervals were 8, 12, and 24 hours. Red lines at 400 mg.hr/L represent the target AUC_24_ ≥400 mg.hr/L with the assumption of the MIC of 1 µg/mL.

The determination of vancomycin dose and dosing intervals was conducted without stratifying the population into specific subgroups based on demographic or clinical factors. This approach evaluates dosing regimens across the entire virtual or observed population, assuming uniform pharmacokinetic behavior and therapeutic targets. **Figure 4** shows simulations of vancomycin concentration–time curves using the full model population (n=10,000; global inclusion). Except for 20 mg/kg/day every 24 hours and 10 mg/kg/day every eight hours in preterm and term neonates, respectively, practically all dose scenarios resulted in around 75% of the population reaching the target AUC_24_ ≥ 400 mg h/L. Preterm infants who received dosage regimens of 20, 30, and 40 mg/kg/day had PTAs of 0.5, 0.85, and 0.96, respectively (**Figure 4a**). PTA levels in term neonates rose from 0.5 at 10 mg/kg/day to 0.75 at 20 mg/kg every 12 hours and 0.80 at 15 mg/kg every 8 hours (**Figure 4b**). Additionally, both groups’ average Cmin, ss were evaluated individually. In term and preterm neonates, respectively, simulations of a 15 mg/kg dose and its dosing intervals of 8 and 12 hours produced average Cmin, ss of 15–20 mg/L (**Supplementary Figure S3**). While Cmin values were explored, the primary focus of this study remains on achieving AUC_24_/MIC targets. However, similarly to **Figure 3**, certain higher-dose regimens result in values exceeding the 600 mg h/L toxicity threshold, highlighting the potential need for dose refinement in specific individuals.

#### 3.4.2 Covariate-based stratification dosing optimization

The reported dosage regimens were further optimized by including variables. After stratifying the population by PMA, GA, and PNA, the simulated doses of 20–60 mg/kg/day found the average AUC_24_ of 552 mg hr/L to be reflected in the overall PTA of the target AUC_24_ of 0.80. Regarding the overall PTA, the PMA-based dosage regimens appeared to offer the most reasonable doses and dosing intervals (**Supplementary Table S2**). The new dosage strategy was optimized by including SCr in the PMA-based dosing regimens from the Neonatal Formulary 7th (Ainsworth, 2014) in light of this conclusion and that of the global inclusion (**section 3.4.1**). The association between the average SCr and PMA was used to determine the cutoff levels for SCr and PMA (**Supplementary Figure S4**). **Table 5** demonstrates that when SCr was added, particularly in the preterm group, the PTA increased. In newborns with PMA 29 weeks and SCr >0.6 mg/dL, the likelihood of achieving the desired AUC_24_ ≥ 400 mg h/L increased from 0.65 to 0.87. **Table 5** shows a thorough comparison of the PTA between the original PMA-based and suggested dosage regimens.

## 4 Discussion

Vancomycin doses in neonatal populations are traditionally based on trough concentrations (Cmin), with target levels ranging from 10–20 mg/L. However, recent studies have shown that Cmin does not consistently correlate with therapeutic efficacy or safety in neonates, especially in relation to nephrotoxicity risk (Janssen et al., 2016). The AUC_24_/MIC ratio is now recognized as a more accurate pharmacodynamic target for vancomycin efficacy, especially in neonates (Tang et al., 2021). This shift in focus has been supported by recent simulation studies, which emphasize the benefits of AUC_24_/MIC-guided dosing strategies for achieving therapeutic efficacy while minimizing nephrotoxicity risks (Alsultan et al., 2023). AUC_24_/MIC ≥ 400 is essential for maximizing therapeutic outcomes (Rybak et al., 2020; Tang et al., 2021). However, achieving efficacy must be balanced with minimizing nephrotoxicity. While the current study focuses on attaining the lower AUC threshold of 400 mg h/L, and emerging evidence suggests that exceeding an AUC of 600 mg h/L may increase the risk of nephrotoxicity in pediatric populations, including neonates (Lestner et al., 2016; Tang et al., 2021), there remains limited prospective data defining a clear toxicity threshold in neonates. In our analysis, we observed a subset of patients exceeding this exposure limit, but the clinical significance of these elevated AUC values in neonates remains unclear. Future studies should focus on prospectively evaluating the relationship between AUC24 exposure and nephrotoxicity incidence in neonates to refine upper exposure limits.

Relying solely on Cmin is insufficient for dose optimization due to rapid developmental changes in neonates. A shift towards AUC_24_/MIC-based dosing is crucial for ensuring therapeutic efficacy and avoiding adverse effects. Vancomycin dosing in neonates, particularly preterm newborns, is complicated by the considerable variation in drug clearance caused by variables such body weight, SCr, PMA, and GA (Pham, 2020; Hughes et al., 2023). Recent work by Hughes et al. (Hughes et al., 2023) underscores the importance of incorporating SCr and maturation covariates into PopPK models to improve predictive performance and optimize neonatal dosing regimens. These differences are not taken into consideration by standardized dose models, which frequently leads to less-than-ideal treatment. Therefore, to take these individual differences into consideration and enhance therapeutic results, customized dosage regimens are required.

With the PTA of AUC_24_/MIC ≥ 400 mg h/L, the model-based dosing strategy of vancomycin in the neonatal population was thoroughly optimized in the current study using modeling and simulation techniques. Additionally, it highlighted the steps taken prior to dose optimization, including simulation of a virtual population of preterm and term infants and review of dosing regimens using a variety of references (Jarugula et al., 2022). To our knowledge, the virtual population created in this study is the most thorough representation of preterm and term neonatal characteristics. Significant factors (weight, SCr, PMA, GA, PNA, and HT), which were collectively taken from clinical cohorts, literature (WHO Multicentre Growth Reference Study Group, 2006; Ceriotti et al., 2008; Boer et al., 2010), and the child growth standards (WHO Multicentre Growth Reference Study Group, 2006), were used to simulate the data. Preterm and term neonates have slightly different data distributions. Due to the few standards that were available for the preterm neonates, the distributions of the simulated variables, for instance, were right skewed in the preterm group while they were normally distributed in the term group. The data distributions, however, were comparable to the references and the relationships between each covariate were substantially preserved. When these simulated data were combined, it created a special environment for studying the neonatal population across the full age range, depicting continuous growth and development from preterm to term babies using patient data and standard references, respectively.

This study adopts a PopPK model to optimize vancomycin dosage schedules for newborns. Important patient-specific factors that are important predictors of vancomycin clearance and distribution are integrated into the model, such as weight, PMA, GA, and SCr. Instead of depending on general population-based assumptions, the model tailors dose regimens based on the distinct characteristics of each newborn by simulating clinical events in a sizable, heterogeneous virtual cohort of neonates (n=10,000). Also, while the bedside Schwartz equation (k=0.413) was retained for consistency with the externally validated popPK model, we acknowledge that neonatal-specific coefficients (k=0.33 for preterm, k=0.45 for term neonates) have been proposed for improved renal function estimation. Future sensitivity analyses should explore whether alternative k-values influence vancomycin clearance predictions and dosing recommendations in neonates. While this study utilized the Bhongsatiern et al. PopPK model, we acknowledge that other published models for vancomycin in neonates exist, some of which do not require HT for eGFR estimation. Including neonatal HT can have drawbacks. When it comes to both quality and quantity, HT is thought to be the least trustworthy measurement for newborns. Neonatal patients at clinics reported measurement errors of between 0.5 and 1 cm in 59% and 34% of cases, respectively. Possible inaccuracies with the sparse HT data could result in extra random mistakes and change the outcomes of our dosing simulation (Johnson et al., 1997; Wood et al., 2013). Also, HT was imputed completely in two of the datasets, and partially in the third dataset. The imputation of HT data, while necessary for CrCL estimation, poses some constraints. Although HT imputation was carefully conducted using statistical methods that ensured consistency with real-world neonatal growth data, we recognize that the reliance on imputed HT introduces a layer of uncertainty in real-world data analysis, which was not explicitly tested in this study. Alternative PopPK models that do not require HT for eGFR estimation may offer a solution to this challenge. Future research should explore the applicability of such models while ensuring they retain predictive accuracy comparable to the selected PopPK model used in this study. For datasets with higher levels of missingness, conducting future sensitivity analyses to evaluate the effect of HT imputation would strengthen the confidence in the proposed dosing strategies.

The U.S. Food and Drug Administration (FDA) strongly advises population pharmacokinetic model predictive performance, particularly when the application is used to establish dose recommendations (U.S. Food and Drug Administration, 2022). Given the variations in patient demographics, vancomycin dosing regimens, and laboratory testing of serum vancomycin and creatinine concentrations, this approach underscored the significance of the published model’s generalizability. In this study, three universities in Belgium, New Zealand, and the US provided external data cohorts for the published model (Bhongsatiern et al., 2015) evaluation. All three cohorts underwent the test satisfactorily, indicating that the presented model is applicable for dosage optimization and is generalized. The VPC plots of the datasets from Salt Lake City and Dunedin show that 10% of the observed data are outside the 90% prediction zone and that there is no biased tendency to overestimate or underestimate the data (**Figure 2**). The mean and variance of the npde were used to identify slightly under- and over-predictions. However, there was no evidence of systematic bias in the population prediction. The published model’s overall robust predictive performance in the datasets from Salt Lake City and Leuven. However, the model did not perform as well in the Dunedin dataset, where the median APE using PRED was 25%, reflecting suboptimal predictions (**Table 3**). The unsatisfactory assessment of the prediction errors may be attributed to variations in the times that blood samples were taken, the observed SCr, and the SCr assay. When compared to the enzymatic approach used in the datasets from Salt Lake City and Leuven vs the dataset from Dunedin, for instance, the Jaffe method utilized in the dataset from Dunedin is known to overstate the SCr in newborns because of their specific matrix (bilirubin, albumin) (Peake and Whiting, 2006; Allegaert et al., 2012). Comparable SCr values were calculated (Srivastava et al., 2009) although this discrepancy may still be due to the elevated SCr in the premature newborns of the dataset from Dunedin. Beyond differences in these mentioned factors, additional population-level differences may have contributed to the lower predictive performance observed in the Dunedin dataset. Compared to Utah and Leuven, the Dunedin cohort consisted of a higher proportion of extremely preterm neonates (PMA < 28 weeks), with lower birth weights and more variability in renal function (SCr distribution). These factors may have impacted the external validation performance, as the published PopPK model was primarily optimized on datasets with a broader range of neonatal maturational stages. Furthermore, while the smaller sample size in Dunedin (n=22) may have influenced predictive variability, it is unlikely to be the sole reason for the differences observed. The differences in demographic characteristics, maturational status, and potential variations in clinical management between centers should be considered in future model refinements.

Red Book^®^ (American Academy of Pediatrics, 2009; American Academy of Pediatrics, 2012; American Academy of Pediatrics, 2021), Neonatal Formulary 6^th^, 7^th^ and 8^th^ Edition (Hey, 2011; Ainsworth, 2014; Duke and William, 2020), and Neofax (Young and Mangum, 2011; Micromedex I. Neofax® Essentials, 2023) dosing regimens were evaluated using the PTA criteria at the AUC_24_/MIC ≥ 400 mg h/L. Given the MIC of 1 g/mL, three out of five dosage regimens achieved 75% probability of the required AUC_24_.

Using the global inclusion approach (no population stratification), AUC_24_/MIC ≥ 400 mg h/L was identified as the target for dose optimization. It was postulated that growth and developmental changes reflected by GA alone (Capparelli et al., 2001) were sufficient to determine a single dose and dosing interval achieving 80% PTA. For preterm and term newborns, daily dosages of 30–40 mg/kg/day and 40–45 mg/kg/day, respectively, provided the best PTA, considering the AUC_24_ target (**Figure 4**).

The study proposes an optimized dosing strategy for vancomycin, aiming to optimize its efficacy in neonates. The primary dosing target is AUC_24_/MIC ≥ 400 mg·hr/L, which maximizes the likelihood of effective treatment for serious infections like MRSA (Frymoyer et al., 2019; Rybak et al., 2020). However, higher dosing in neonates can lead to nephrotoxicity. The study uses AUC_24_/MIC as the primary dosing target, balancing efficacy and safety. Patient-specific factors like PMA, GA, SCr, and weight are incorporated into tailor vancomycin exposure to individual needs. This approach reduces the likelihood of overexposure in neonates with lower renal clearance and minimizes nephrotoxicity. Optimized dosing also helps avoid underdosing in larger or more mature neonates, ensuring therapeutic efficacy without excessive risk of side effects. Additionally, while the log-linear trapezoidal method was used for AUC calculations in this study, future investigations could explore direct integration from the model as an alternative approach. Direct integration may offer additional precision under certain conditions, though its computational feasibility and adaptability across varying dosing regimens should be assessed before widespread implementation.

In comparison to preterm newborns, optimizing doses and dosing intervals for term neonates is comparatively easier, particularly during the first week of life, due to their more predictable pharmacokinetic profiles. We hypothesize that incorporating SCr into dosing adjustments captures the developmental changes in neonatal kidney function while conservatively preventing unnecessary dose escalation. However, further research is required to establish the importance of the upper AUC threshold (e.g., < 600 mg h/L) and its relation to nephrotoxicity in this sub-population of neonates (Lestner et al., 2016). Additionally, recent studies highlight the need for defining nephrotoxicity thresholds specific to neonates, which remain a critical gap in vancomycin pharmacotherapy research (Tang et al., 2021; Alsultan et al., 2023).

Recent advancements in precision dosing software have facilitated real-time individualized dosing adjustments, reducing reliance on static dosing schemes. However, such tools are only as effective as the pharmacokinetic models that inform them. The present study’s findings contribute to this growing field by providing an externally validated PopPK model and optimized dosing recommendations that can serve as the foundation for precision dosing algorithms. Additionally, many clinical settings, particularly in resource-limited regions, lack access to sophisticated dosing software, making validated population-based dosing strategies a crucial clinical tool. Future research should focus on integrating these findings into clinical decision support tools to enhance accessibility and real-world applicability.

### 4.1 Limitations

This study has several limitations. First, the lack of information on individuals with SCr levels more than 0.6 mg/dL. The dose optimization for this group could not be evaluated because the term neonates was created using SCr data sources that excluded kidney damage and associated renal insufficiency. Furthermore, this study does not evaluate the implications of higher AUC values on nephrotoxicity. Although evidence in older pediatric populations indicates risks associated with AUC_24_ values > 600 mg h/L, such thresholds are not yet well-defined for neonates. The use of the published model in the dosing simulations constituted a second restriction. Even if the model was successfully validated, it is crucial to assess the generalizability of the model cautiously when there is a clear disparity in the patients’ demographics. Incomplete or inconsistent covariate information is an often limitation in retrospective studies. The retrospective and additional multicentric design of this study also introduces variability in sampling schedules and bioanalytical methods, among other factors, potentially contributing to additional unexplained variability. Additionally, vancomycin preparation may differ across centers, with hospital pharmacies managing dosing in some locations while ward nurses preparing doses in others. While all centers adhered to standard dilution and administration protocols, minor procedural variations may have influenced drug exposure. Finally, the results found in this study should be validated in a prospective clinical study. Another limitation is the lack of representation of neonates from resource-limited settings. Differences in healthcare infrastructure, nutritional status, and neonatal comorbidities could influence vancomycin pharmacokinetics, potentially limiting the generalizability of the findings to these populations.

### 4.2 Future research

The study uses a novel approach by generating a virtual neonatal population, allowing for a robust simulation of various clinical conditions across a wide range of neonatal characteristics. This method enhances the generalizability of the findings, ensuring that the optimized dosing strategies are applicable across different neonatal subgroups, including those with varying degrees of renal function. The integration of SCr, a marker of renal function, into the dosing model represents a significant advancement over traditional models that often do not account for this key covariate. For neonates experience changes in renal function, the inclusion of SCr in dosing calculations allows for more accurate predictions of vancomycin clearance, ensuring that dosing is tailored to the specific renal capacity of each neonate. The inclusion of GA and PMA allows the model to adjust dosing regimens dynamically, accounting for the neonate’s developmental stage (De Cock et al., 2014). Weight, as a determinant of volume of distribution, is crucial in calculating individualized loading and maintenance doses. These factors, when combined, enable a precise, tailored dosing approach that can be adjusted as the neonate develops. Although the population pharmacokinetic model was validated using external datasets, additional prospective clinical studies are needed to fully confirm the safety and efficacy of the proposed dosing regimens in real-world settings. Future studies should focus on further refining vancomycin dosing strategies by incorporating real-world data on AUC_24_ and acute kidney injury (AKI) incidence in neonates. Evaluating the relationship between AUC_24_ exposure and nephrotoxicity risk could provide stronger evidence for defining an upper safety threshold for neonatal dosing. Prospective data collection and model-based analysis of AKI rates across multiple clinical sites would further enhance the clinical applicability of PopPK-guided dosing recommendations. Additionally, while an AUC threshold of >600 mg h/L has been associated with nephrotoxicity in adults, it remains unclear how this translates to neonates. Future prospective studies should assess the correlation between AUC_24_ and nephrotoxicity risk in this population. This includes establishing upper AUC thresholds to guide dosing strategies that optimize both efficacy and safety, minimizing the risk of vancomycin-induced nephrotoxicity in neonates. The existence of both vancomycin toxicity and efficacy targets would help optimize antibiotic dosing strategies in neonates. Future research should also aim to validate these findings in resource-limited settings, where differences in neonatal characteristics and healthcare practices may necessitate adjustments to the proposed dosing strategies.

### 4.3 Summary

The study simulated the effects of vancomycin dosing regimens on both preterm and term neonates, providing a comprehensive framework for testing these regimens. The model accounted for rapid physiological changes in neonates, such as renal maturation, body weight, and drug distribution volumes. The study found that renal function improves with increasing GA and PMA, impacting vancomycin clearance. By incorporating these developmental factors, the study was able to optimize dosing intervals more accurately. Preterm neonates with slower renal clearance required longer intervals to avoid drug accumulation, while term neonates with more mature renal function could tolerate shorter intervals without risking toxicity. The simulations showed that dosing regimens could be adjusted to reflect the neonate’s developmental stage, with preterm neonates with lower renal function needing a dosing interval of 12–24 hours to achieve AUC_24_/MIC ≥ 400 mg h/L. In summary, this study addresses the critical need for optimized vancomycin dosing in neonates by moving beyond traditional Cmin-based approaches and utilizing AUC_24_/MIC optimization through population pharmacokinetics. The methodology’s integration of developmental and clinical covariates, including PMA, GA, SCr, and weight, provides a robust framework for individualized therapy, ensuring both efficacy and safety in neonatal care.

## 5 Conclusion

This study demonstrates the effectiveness of a population pharmacokinetic (PopPK)-based optimized dosing strategy for vancomycin in neonates. By incorporating developmental factors such as PMA, SCr, and weight, the model achieved an 80% probability of target attainment (PTA) for the therapeutic AUC_24_/MIC ≥ 400 mg·hr/L. The use of a virtual neonatal population enabled robust validation and dosing optimization across diverse clinical scenarios, ensuring broad applicability to both preterm and term neonates.

The findings highlight the limitations of traditional Cmin based dosing approaches and emphasize the need for AUC_24_/MIC-guided strategies to improve therapeutic efficacy. Furthermore, the integration of WHO growth standards and rigorous data imputation ensures that the proposed regimens are clinically relevant and adaptable to various healthcare settings. This optimized approach to vancomycin dosing marks a significant advancement in neonatal pharmacotherapy, providing a scalable framework for individualized care and setting the stage for further research to confirm safety and efficacy in real-world practice.

## Data Availability

The raw data supporting the conclusions of this article will be made available by the authors, without undue reservation.
