# Supplementary material for: Vancomycin dosing in neonates: enhancing outcomes using population pharmacokinetics and simulation
Source: Front Antibiot. 2025 May 8;4:1568931. doi: 10.3389/frabi.2025.1568931 (PMC12095254; doi:10.3389/frabi.2025.1568931)
Supplement: Supplementary file 1 [file DataSheet1.docx]

**Supplemental Tables**

**Table S1 Statistical summary of virtual neonatal population (n=10,000)**

| **Covariates** | **Preterm (n=5,000)** | | | **Term (n=5,000)** | | |
| --- | --- | --- | --- | --- | --- | --- |
|  | **Mean ±SD** | **Median (range)** | **Mode** | **Mean ±SD** | **Median (range)** | **Mode** |
| Weight (kg) | 1.7±0.92 | 1.4  (0.41-4.8) | 0.75 | 3.7±0.70 | 3.7  (1.4-6.5) | 3.1 |
| Serum creatinine concentration (mg/dL) | 0.55±0.23 | 0.51  (0.11-1.5) | 0.47 | 0.34±0.08 | 0.34  (0.10-0.73) | 0.34 |
| Postmenstrual age (weeks) | 32.1±5.0 | 31.4  (23.0-44.0) | 32.7 | 40.7±1.50 | 40.7  (37.5-44.0) | 40.6 |
| Gestational age (weeks) | 27.8±3.4 | 27.0  (22.0-36.0) | 25.0 | 38.6±1.10 | 39.0  (37.0-41.0) | 39.0 |
| Postnatal age (days) | 30.0±20.7 | 25.0  (4.0-115.0) | 10.0 | 14.4±6.80 | 14.0  (4.0-28.0) | 5.0 |
| Body length (cm) | 38.2±7.6 | 37.6  (15.3-61.0) | 34.3 | 51.8±7.60 | 51.9  (36.0-68.9) | 37.6 |

**Table S2 Optimization of doses and dosing intervals using hypothetical population stratified by PMA, PNA, and GA**

| **Population characteristics** | | **Dose (mg/kg)** | **Interval (hrs.)** | **C_min_ (mg/L)** | **AUC_24_ (mg.hr/L)** | **Probability of target attainment** |
| --- | --- | --- | --- | --- | --- | --- |
| **PMA (weeks)** | **PNA (days)** |  |  |  |  | **AUC_24_ ≥400** |
| GA <37 | - | 10  10  15  20  20  30  40 | 8  12  12  12  24  24  24 | 20.31±10.2  11.45±6.4  17.21±9.7  22.85±12.6  6.85±5.1  10.28±7.8  13.82±10.6 | 640.06±257.0  426.25±168.1  641.22±257.8  851.64±337.9  404.89±168.8  636.92±253.6  855.03±343.7 | 0.85  0.49  0.85  0.96  0.49  0.85  0.97 |
| GA <37 | <7 | 10  10  15  20  20  30  40 | 8  12  12  12  24  24  24 | 22.43±12.0  13.00±7.6  19.29±11.3  25.26±14.4  8.28±6.9  12.19±10.1  16.13±12.6 | 691.33±301.6  463.1±199.8  689.70±294.0  912.48±380.7  471.04±208.3  694.51±311.8  923.19±391.9 | 0.87  0.57  0.86  0.98  0.56  0.88  0.97 |
| GA <37 | ≥7 | 10  10  15  20  20  30  40 | 8  12  12  12  24  24  24 | 18.20±9.5  10.22±6.1  15.37±9.2  20.32±12.0  6.03±4.9  8.92±7.3  11.97±9.9 | 588.99±240.3  393.87±163.0  592.81±243.6  785.58±321.9  397.53±166.4  593.51±244.8  789.67±330.5 | 0.78  0.40  0.78  0.93  0.42  0.78  0.93 |
| GA ≥37 | - | 10  15  20  10  15  20  30  20  30  40 | 8  8  8  12  12  12  12  24  24  24 | 10.32±4.6  15.47±7.0  20.60±9.4  5.22±2.9  7.77±4.2  10.38±5.6  15.49±8.3  2.20±1.9  3.26±2.8  4.40±3.8 | 393.79±126.4  590.3±189.0  786.8±257.7  262.9±85.6  392.60±126.0  523.93±167.9  784.03±247.8  262.25±85.1  393.03±125.3  526.40±171.5 | 0.41  0.86  0.98  0.07  0.41  0.76  0.98  0.07  0.41  0.77 |
| GA ≥37 | <7 | 10  15  20  10  15  20  30  20  30  40 | 8  8  8  12  12  12  12  24  24  24 | 12.47±4.4  18.75±6.8  25.41±9.1  6.59±2.8  9.83±4.3  13.05±5.6  19.64±8.2  3.05±1.9  4.65±3.0  6.09±4.0 | 450.65±119.0  671.94±187.4  912.04±252.8  300.32±83.8  448.17±127.0  604.45±170.8  904.19±248.0  300.98±81.1  451.46±129.6  604.48±168.1 | 0.64  0.96  0.99  0.12  0.60  0.90  1.00  0.11  0.60  0.92 |
| GA ≥37 | ≥7 | 10  15  20  10  15  20  30  20  30  40 | 8  8  8  12  12  12  12  24  24  24 | 9.36±3.8  14.04±5.7  18.91±7.7  4.65±2.3  6.92±3.4  9.21±4.5  13.8±6.8  1.78±1.4  2.71±2.1  3.60±2.8 | 369.82±107.8  552.58±160.4  744.01±216.6  247.63±73.8  369.21±107.6  491.91±140.7  736.01±213.5  245.74±70.2  371.92±108.4  494.32±144.0 | 0.34  0.84  0.97  0.03  0.34  0.72  0.98  0.03  0.34  0.72 |

Reported values of C_min_ and AUC_24_ are in mean (±SD). Probability of target attainment is from 0 to 1, where 1.00 is the highest probability. PMA, postmenstrual age (weeks); GA, gestational age (weeks); PNA, postnatal age (days); SCr, serum creatinine concentration (mg/dL); C_min_, minimum concentration at steady state (mg/L); AUC_24_, area under the concentration-time curve at steady state (mg.hr/L); AUC_24_≥ 400, probability of achieving the AUC_24_ of ≥ 400 mg.hr/L

**Supplemental Figure Legend**

**Fig. S1 Correlation of body length (HT) and postmenstrual age (PMA) in the observed and imputed datasets**

Comparison of the data distribution between observed and imputed body length (HT, cm). Median (interquartile range [IQR]) of the observed data from Dataset 1 (n=403) was 41.0 (35-48) cm, imputed Dataset 2 (n=185; median = 40.9 [35.9-47.5]) cm, and Dataset 3 (n=22; median = 33.5 [31.8-36.5]) cm. Positive correlation of HT and postmenstrual age (PMA, weeks) was observed.

* Dataset 1: Salt Lake City, USA; Dataset 2: Leuven, Belgium; Dataset 3: Dunedin, New Zealand.

**Fig. S2 Goodness-of-fit plots of the evaluated model. Population predictions (PRED) (left) and individual predictions (IPRED) (right) versus observations (DV).** The identity line (dashed black) represents the ideal scenario where observed and predicted values align perfectly. The distribution of data points illustrates the model's ability to predict population-level pharmacokinetics.

**Fig. S3 Simulations of the average concentration-time profile using vancomycin doses and dosing intervals**

Scatterplots of average concentrations and time simulated from different doses and dosing intervals of vancomycin intermittent IV infusion using an entire age range of the hypothetical population. Dosing intervals (8, 12, and 24) were specified as titles of each plot. Vancomycin doses (mg/kg) were 10 (dashed lines), 15 (solid lines), 20 (dot-dashed lines), 30 (two dot-dashed lines), and 40 (dotted lines). Two red lines at 15 and 20 mg/L are shown to depict the range of target therapeutic concentrations.

**Fig. S4 Correlation between average serum creatinine concentrations (mg/dL) and postmenstrual age (weeks)**

Correlation between serum creatinine (SCr) and neonatal age (PMA, GA, and PNA) was determined to understand SCr levels at different ages of the hypothetical neonatal population. As shown in Figure S2, a rise of the average SCr (0.6-1.1 mg/dL) was observed at the PMA of 23-29 weeks, and the SCr decreased from 0.6 to 0.4 mg/dL during the PMA of 30-36 weeks. At the PMA of ≥ 37 weeks, the average SCr were relatively stable (0.4-0.3 mg/dL)
