# Supplementary figures and images for: Vancomycin dosing in neonates: enhancing outcomes using population pharmacokinetics and simulation
Source: Front Antibiot. 2025 May 8;4:1568931. doi: 10.3389/frabi.2025.1568931 (PMC12095254; doi:10.3389/frabi.2025.1568931)

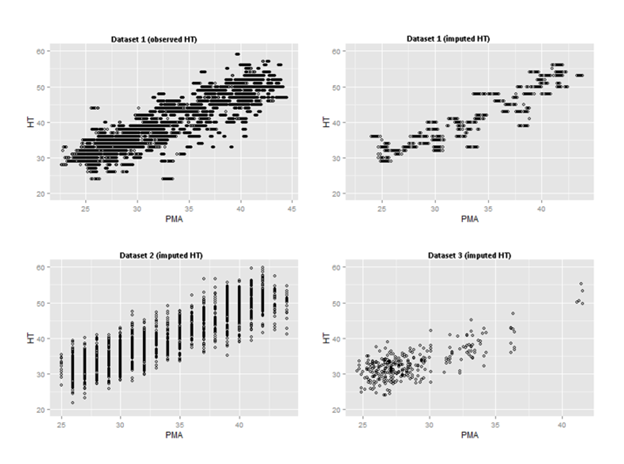

Supplement: Supplementary file 2 [file Image1.tif]

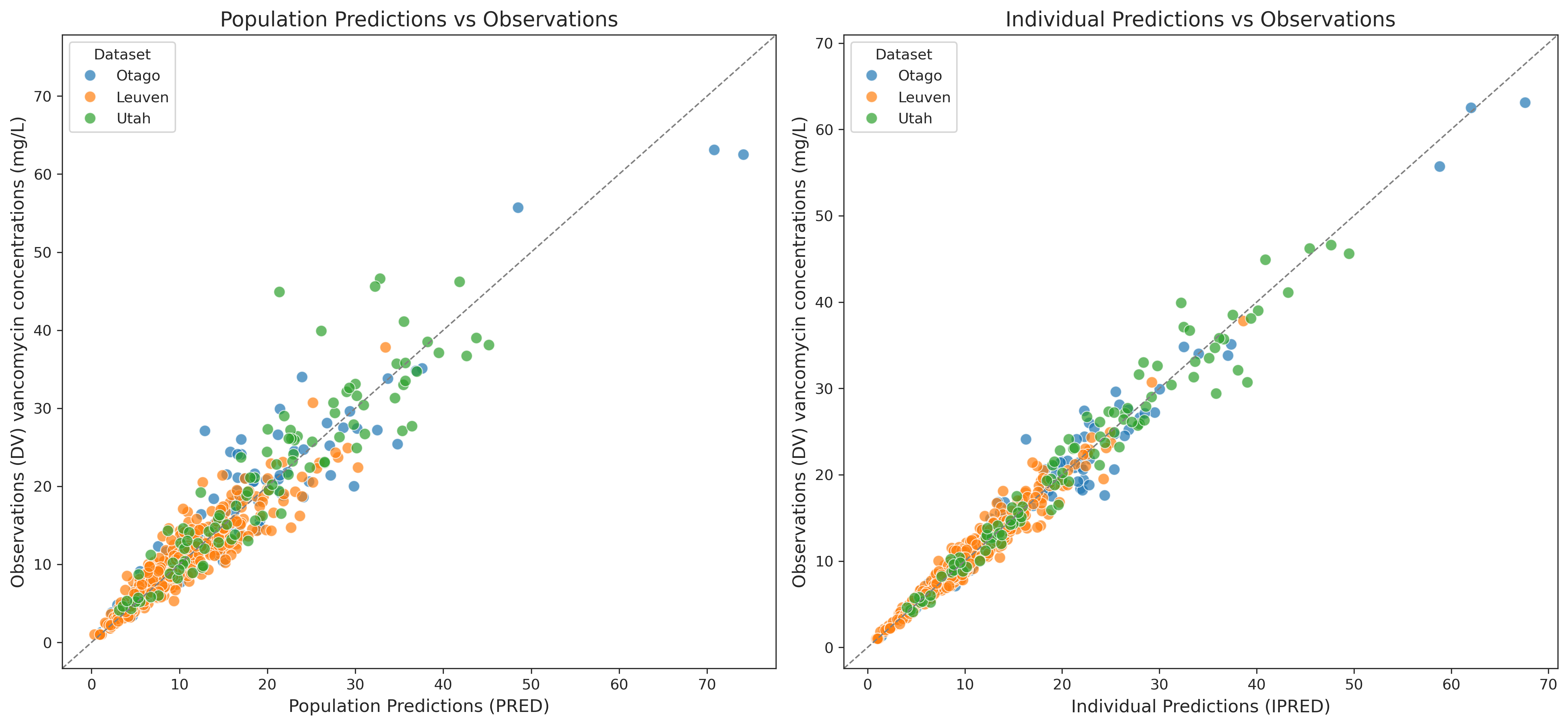

Supplement: Supplementary file 3 [file Image2.png]

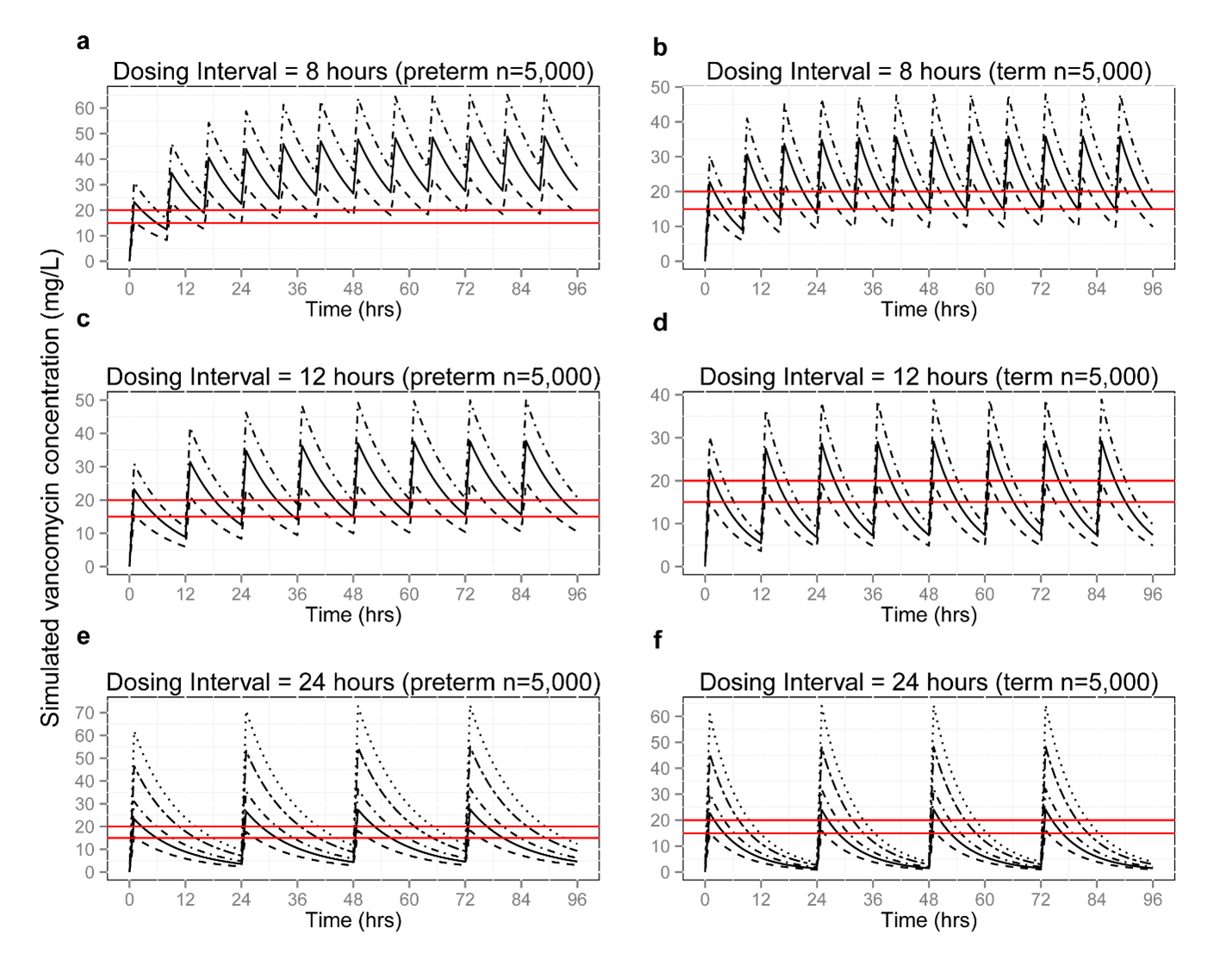

Supplement: Supplementary file 4 [file Image3.tif]

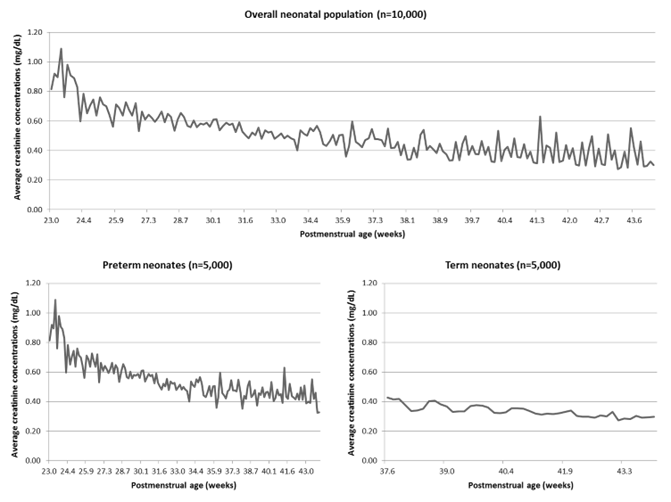

Supplement: Supplementary file 5 [file Image4.tif]
